# Understanding the role of invictus games in supporting the transition for UK disabled military veterans

**DOI:** 10.3389/fspor.2025.1609967

**Published:** 2025-09-23

**Authors:** Chris Mackintosh, Niamh Reavill, Rebecca O'Hanlon, Dan Roberts

**Affiliations:** ^1^ Department of Sport and Exercise Sciences, Manchester Institute of Sport, Manchester Metropolitan University, Manchester, United Kingdom; ^2^ Department of Sport, Exercise and Health, University of the West of Scotland, Paisley, United Kingdom; ^3^ MCR Active, Manchester, United Kingdom

**Keywords:** sport, military, invictus games, physical activity, narrative analysis

## Abstract

This study explores the experiences of veterans with disabilities regarding how sport and physical activity (PA) impacted their transition into civilian life. The study sought to understand how veterans' experiences since injury affect their well-being, independence, and future. Eight veterans (five women) with a physical disability were recruited via the Wounded, Injured and Sick (WIS) Invictus Games Foundation page. The qualitative semi-structured interviews studied their experience in sport and PA and transitioning out of the Armed Forces. Data were analysed using narrative analysis. Caddick's theoretical framework was used to explore and analyse the presence of five core veteran narratives in distinct narrative types; “*struggling against decline*”, “*minimization*”, “*victimhood*”, “*life-as-normal*” and “*dramatic overcoming*”. A further novel narrative was also heard, referred to as type, which was previously absent, emerged in the data, due to the age and competitiveness of the participants. This novel narrative, was referred to as “*getting on with it*” *and* was also found to be prominent in the data. The study provides insight into the experiences of veterans with disabilities and the role of sport and PA in their transition into civilian life. The findings suggest that promoting sport and PA and providing opportunities for participation in sporting events can be an effective tool in supporting veterans, providing recommendations for future work around disabled veterans engagement in sport and physical activity, specifically Invictus Games (IG) global event movement.

## Introduction

This paper will explore how engaging in sport and physical activity (PA) can impact physically disabled veterans who have left the Armed Forces and emphasise the importance of maintaining independence for veterans with disabilities. The use of sport and PA as a rehabilitation tool for Wounded, Injured or Sick (WIS) military veterans is not a new or recent phenomenon. Sir Ludwig Guttman, a doctor at Stoke Mandeville hospital, opened the first ever spinal injuries unit working with soldiers injured in the Second World War. Guttman recognised the physiological and psychological values of sport and PA, and introduced this as part of the rehabilitation, establishing the Stoke Mandeville Games. These Games set the foundation for the Paralympic Games and, more recently, the Invictus Games (IG) (1, 2). While both competitions focus on athletes with disabilities, the Invictus Games (IG) is specific to service personnel and uses sport and PA as a tool for rehabilitation and to foster understanding and respect for the military community (3). Wider literature has acknowledged this distinctive focus on military personnel, as they can face unique and complex challenges when transitioning from the military, such as social isolation, poor mental health, unemployment, and homelessness, which are further exacerbated when individuals have a disability or impairment due to their service (2, 4–10). The paper is a real first to explore the IG athletes in this study from a military sociology perspective using narrative analysis embedded within the wider literature of sport-for-development and the role of this to address social change for this population group in the UK. We believe it has relevance to many IG participants beyond the UK, but also military veteran contexts where there are a growing number of schemes using sport and PA to address social isolation, societal transition and ex-military and military identity and welfare (11). Other authors have explored in rich detail the process, identity impact and narratives that could be drawn from military identity to civilian transition (12–14). Grimmell (15) for example has specifically examined dialogical self theory and what he refers to as “aborted transitions”. Whilst no mention is made of the role of sport and physical activity in this process, it offers a powerful substantive context for this paper. Again, further related work has also usefully explored PTSD in Swedish veterans and their cultures as wounded warriors in a nation of peace (16). Moral and physical, alongside national and social identities are interwoven here within military identities.

As already stated, there is extensive literature on psychological and social psychology theory and military identity [see (16)]. In this body of work military identity is seen as a contested and fluid dialogue between authors in this space (16–18). Due to limitations and focus in this specific paper, we signpost readers in this domain to engage with what Grimmell (16) suggests as the vital analysis of,

“Depersonalization and self-verification highlight the identification with a category (stronger emphasis in social identity theory) and the behaviours associated with the group (stronger emphasis in identity theory)—and both are important to understand identity processes among veterans”.

**Figure 1:**
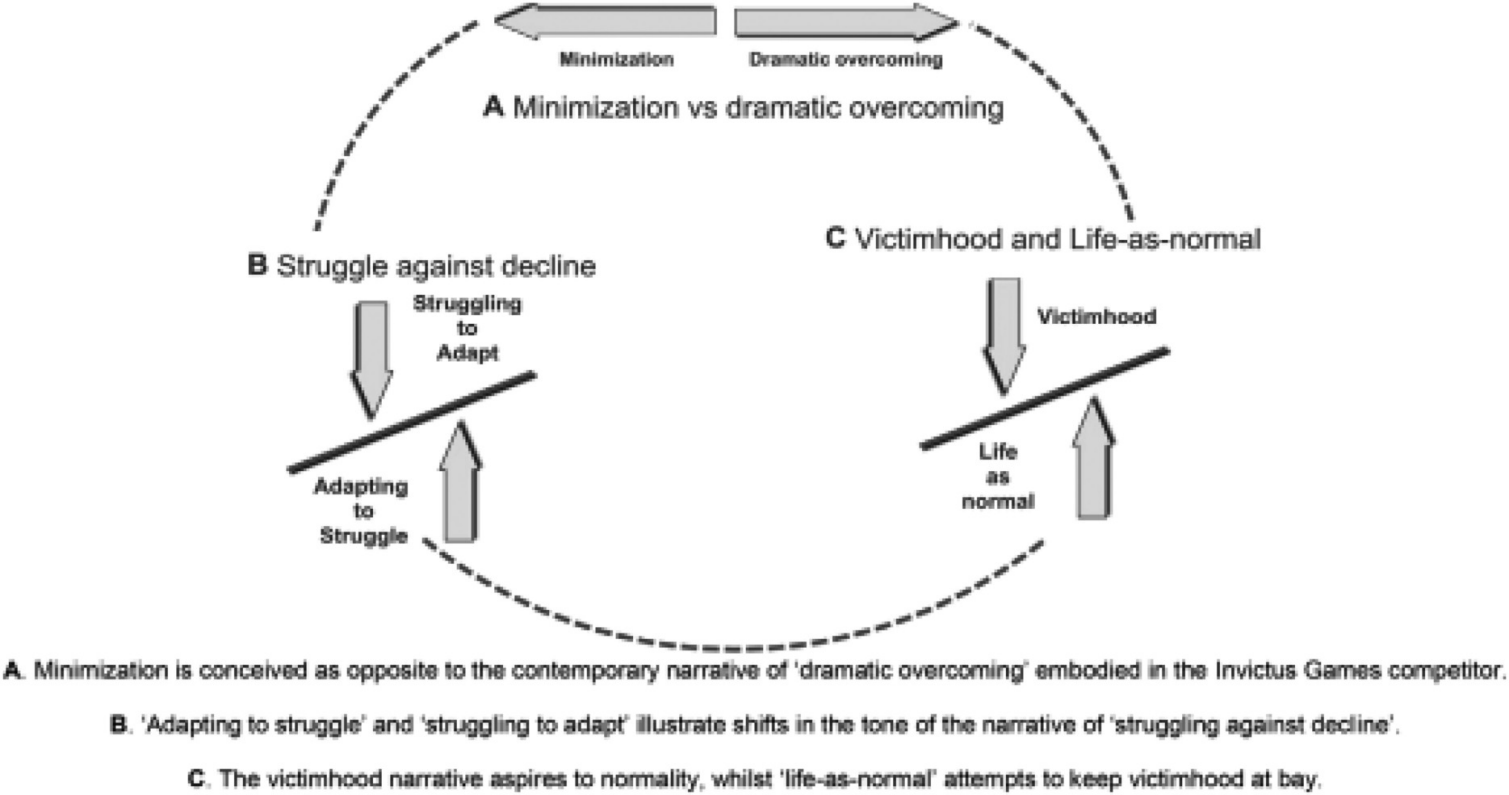
Conceptual model of limbless veterans’ narratives (6).

In our case, using a theory framework by Caddick et al. (6), (Figure 1) itself is a rare example of an attempt to use a more sociological conceptualisation in this project where we apply the five main often inter-related types of strategies and identities for transition through sport and PA and extend this model with a new novel sixth narrative category for the IG disabled athlete veterans of “*getting on with it*”.

In the United Kingdom (UK), a veteran is defined as all personnel who have served more than one day, inclusive of their dependents (19), Despite this definition, stereotypes continue to persist, particularly around identity dimensions such as age, gender, ethnicity, disability and class (20). In this paper, our novel focus on young and female veterans seeks to disrupt and disturb these stereotypes. Previous literature has established that young veterans especially face challenges, in relation cultural adaptation and transitioning into the civilian environment, that are less prevalent among their older peers (8, 21). Likewise, young and/or female veterans often have a family to support while undertaking their transition and, when faced with under- or un-employed, insufficient income, relocation and housing issues, and changes in healthcare, this can contribute additional pressure and difficulties (22). Thus, attending to military specific challenges, alongside the broader life challenges evident among civilian populations, this paper offers a valuable contribution extending previous literature that has examined these military specific and civilian challenges in isolation.

Much of the existing literature examining the transition into civilian life adopts a psychological perspective, and this understanding is now well-established (23). However, more recently, there has been a shift from traumatic transition narratives to adopting a broader focus on the range of individual transition experiences (24). Sport and PA has featured in these discussions, as a tool for rehabilitation, support and integration, yet the explicit connection and intersection between the military to civilian transition and sport for development (SfD) is yet to be made (25). Situated within this context, this paper is novel in its focus on the intersection of SfD specific to the Invictus Games, physical disability and younger military veteran transition, offering valuable contributions to the aligned fields of military and SfD research. This paper aims to examine the responses to physical disabilities by military veterans, understand how sport and PA has contributed to this, and critically evaluate the impact of these responses to shape an understanding of types of strategies they develop. The literature review covers the history and context of disabled veterans, the definition of disability and the role of sport and PA in military transition.

### The veteran and policy context of the UK

The purpose of this section is to outline how the Armed Forces of Great Britain can be traced back to the Acts of Union in 1707 when the Armed Forces of England and Scotland were amalgamated (26). Since the formation, the UK military has been involved in conflicts in 171 countries around the world (27). Furthermore this section illustrates how although figures vary, an estimate of between 136.5 and 148.5 million people died in wars and conflicts during the 20th Century. Of these, 13–15 million deaths were in World War I, and 65–75 million civilians and military personnel died in World War II (28). Alongside these figures, over 370,000 British military personnel were injured during WWII (29). Whilst Invictus is a global Games event for military veterans all participants in this study are from an ex-UK military context.

We aim to give context to the scale, scope, and size of this veteran sector in the UK using most recent government statistics (30) which report 1,568,945 full-time England and Wales ex-Service Personnel (as of April 2023). Most personnel were within the Army (56%) and the other 44% split evenly across the Royal Air Force (RAF) and the Royal Navy/Royal Marines (RN/RM). Whilst 14,630 people left the UK Armed Forces, 13,350 new personnel have joined over the past year. This is an increase of 19% more people leaving the Services than in the previous 12 months; this agrees with the trends shown in Figure 1 (31), that the key reasons for people leaving the military are; administrative (disciplinary/temperamental unsuitability), family related and medical reasons. Of the medical discharges between 2001 and 2014, 60% were for musculoskeletal injuries (32). There is currently no agreed definition for the military to civilian transition. Elnitsky et al. (33) suggest that it is best understood as the time period or process of moving from the military into civilian life, meaning that this can vary greatly between individuals. Notably, there is also no established start and/or end points for this transition, therefore a transition can start and end at different points depending on the individual, accounting for the noted variation in the length of time the transition may take.

As of January 2022, the updated *Veterans' Strategy Action Plan* aimed to provide additional benefits for UK veterans, including higher priority for housing, priority care for service-related conditions and access to specific support plans for mental health and prosthetics (34). As part of this, the NHS will be spending £18 million on veteran's healthcare. Additionally, there is an Armed Forces Pension Scheme (AFPS). This specific AFPS is an important financial component of the transition aspect, but we argue needs to be considered alongside socio-cultural aspects we explore in this paper for disabled veterans. For example, recent studies have found that transitioning out of the Armed Forces can be significantly harder for people with a physical disability (6, 8) regardless of the AFPS. The sense of purpose and belonging that the veteran previously had in the military feels as though it has been taken from them and they are required to start a new life outside of the Armed Forces.

### Defining and conceptualising disability

In the following section the definition of disability is explored and explained in a domain we consider highly socio-cultural, physically and politically contested. The Equality Act (35) classes disability as “physical and mental impairment that has a “substantial” and “long-term” negative effect on the ability to do normal daily activities”. Since then, the UN Convention on the Rights of Persons with Disabilities has disregarded this description, recognising that disability is an “evolving concept” and should not have one set definition (36). More recently, models have aided our ability to conceptualise and therefore define disability. The medical phenomenon narrowly focuses on the person's impairment and limitations as a disabled person (37–39), rather than personal and social needs (40). It aims to “fix” the person, physically, with medicine or rehabilitation to reach normality. The charity approach is run side by side with this. Funding aids include additional facilities such as rehabilitation centres but have no power over people's lives as cited in the Convention on the Rights of Persons with Disabilities (36). The social approach contrasts this and concludes that disability is found to be a result of the individual's dealings with an environment. Consequently, the resultant inequality is not due to the impairment of the individual, but because of societal injustice (41), leading to isolation and exclusion towards disabilities (42). Disability is a consequence of societal interaction in personal and environmental factors, not a “mistake” (43).

### The role of sport and physical activity in military transition

The use of sport and PA as a rehabilitation tool for WIS military veterans, and support mechanism for those undertaking the military to civilian transition, is not a new or recent phenomenon. In this section we suggest there is a pre-existing wealth of existing literature that highlights how sport and PA can have a positive influence on many aspects of veterans' lives, including providing enjoyable experiences, supporting personal development and growth, fostering social integration and enhancing physical and mental health (5, 44–46). In Caddick and Smith's (47) systematic review of literature examining the impact of sport and PA on veteran wellbeing, they noted a difference in the types of sport or PA being used, dependent on the type of veteran being targeted and the overall objective of the given programme or initiative. For veterans with a disability or physical injury, it was identified that adapted activities and competitive sport were the most common and effective approach (47). It is suggested that partaking in competitive sports encouraged veterans to accept their disability or injury, rather than being defined by it, and think more positively towards it, contributing both psychological and social benefits (4, 9, 48). Furthermore, being immersed in a competitive sporting environment also supported veterans to develop social connections, finding commonalities with peers that led to increasing confidence and feeling supported as they negotiate their life change, which include a physical injury or disability from service (1, 9, 48).

In addition to competitive sport, being faced with a physical challenge was also seen to motivate those with a physical injury or disability. A study by Burke and Utley (4) explored four injured veterans' stories of climbing Mt. Kilimanjaro and found that being able to accomplish more than initially expected, upon first sustaining the injury, led to a profound sense of achievement and determination. Much like the phenomenological lens espoused by Hawkins et al. (44), the self-determination theory (SDT) (49) was used as a theoretical framework to understand the autonomy, competence, and relatedness of the veterans' experience. However, this has not been explored in depth within the paradigms of sport and PA. Most recently a study was undertaken examining Team Ukraine veterans and the use of an international sporting event for veterans living with disabilities and injured active-duty personnel, led by the United States *Department of Defense Warrior Games* (50). But the Invictus Games, as a far wider larger global event, has not been examined in detail.

Despite these noteworthy impacts and benefits of sport and PA, Caddick and Smith (47) also warn that sport and PA are not a universal remedy for improving veterans lives overall, suggesting that a balanced model of rehabilitation is necessary, encompassing broader factors such as employment, finance, and future planning. This is a sentiment echoed in the SfD literature, which highlights that sport and PA are not the necessary factors for key personal and developmental changes to occur (51, 52). Instead, it is often the mechanisms, processes, and experiences that surround sport and PA that foster changes, and this is a notion that much of the existing literature on the IG points towards (3, 7).

### The invictus games

Only in very recent studies has it started to be explored that the IG plays an important role in assisting veterans with an injury or disability in their recovery, using sport and PA as a mechanism to support them to navigate their life change (2, 3, 7). The 2014 emergence of IG also marks monumental shift in the cultural representation of WIS veterans, as depicted through the lens of sports and PA (53). Through this platform, WIS military personnel are portrayed in a way that challenges the conventional image of the powerful and heroic military and instead highlights complex political narratives of overcoming and redemption (2, 7). The IG narrative encourages us to feel pride, sentimentality, and respect towards disabled veterans who have overcome significant obstacles and promotes the “recovery” aspect. After experiencing traumatic injuries, engaging in sport and PA can give veterans a new sense of direction and purpose. Thus, allowing focus on ability and achievement, whilst providing an avenue for social bonding and camaraderie (7). However, Ives et al. (54) suggest that, when promoting sport and PA to people with disabilities, there should be less of a focal point on 'sport' and more of a push for “activity”, thus, placing an emphasis on the leisure and social aspects. Thus, marking a notable difference between the IG and the Paralympic games, where the IG places greater emphasis on community, support and belonging alongside participation in competitive sport.

Similarly, Caddick et al. (5) found that correspondent with limbless veterans' age, there was an empirical focus on narrating the minimization limb-loss, rather than the triumphs of sporting achievements. This is likely due to younger veterans being the focus of these successes. The main theme throughout focused on maintaining independence, alongside a narrative typology which included four recurring types (struggling against decline, minimization, victimhood and life-as-normal) as a primary emphasis. However, there was absent data when exploring the fifth type of “dramatic overcoming”. This may be culturally prominent in initiatives such as IG, for younger veterans.

### Transitioning with a disability

In this section we signpost the reader to the notion that for those transitioning from the military, with a disability or injury acquired from their service, this can present more significant and complex transition-related challenges. The sense of purpose and belonging that a veteran previously has as a part of the military requires adjustment and new “rules” (5, 6, 8, 12, 13, 55). Disability is a contested concept, and the definition of disability has evolved over time, with the medical and social models aiding its conceptualisation. The recent *Veterans' Strategy Plan* aims to provide additional benefits for the UK veterans, including higher priority for housing, priority care for service-related conditions, and access to specific support plans for mental health and prosthetics. However, transitioning out of the Armed Forces can be particularly difficult for people with physical disabilities. Overall, the UK military continues to play a vital role in global security, and it is essential to provide adequate support and resources for veterans who have served their country. Further exploration was recommended (6) to conduct additional research to better comprehend the militarised narratives that were not discussed in their study due to the ageing-population sample. They suggested exploring how the “dramatic overcoming” narrative is investigated through other initiatives such as the IG, to aid younger veterans in surmounting the challenges of physical disabilities and transitioning out of the military. It is this agenda that this paper seeks to meet.

## Method

The qualitative study utilised an in-depth interview approach to explore the experiences of physically disabled veterans who have engaged in sport and PA, either currently or in the past, who have transitioned out of the Armed Forces in the UK.

### Participants

Participants were recruited via the WIS Invictus Games Foundation Noticeboard through the researcher's gatekeepers. Eight participants (five female and three male) were interviewed. The criteria necessary for each participant was: (a) over 18 years old; (b) have a physical impairment; (c) participate/previously participated in a IG sport or PA; (d) have transitioned out of the military.

### Interviews

Following ethical approval from a University Faculty ethics committee, informed consent was obtained, and a mutually convenient time was scheduled for the participants' MS Teams interview. Face-to-face interviews would have been preferrable to build rapport for the participant, however, after taking disability, mobility, and geographical dispersion into account, this was not possible. These interviews ranged from 45 to 115 min in duration, allowing the researcher to establish a rich, textured and layered rapport and gain insight into the lives of the veterans. Semi-structured interviews facilitated the researcher to gain additional depth into an understanding of the participants “lived experiences” by exchanging open-ended dialogue (56, 57). This allowed for opinions, concepts, and ideas throughout the interview, yet encouraged new articulated answers and feelings (58) to provide richer data. In some cases, due to anonymity and identifiability we have not included full details but the details the participants felt comfortable with us mentioning. Interview topics used were semi-structured in nature, but allowed for discussion around threads of stories, life events and experiences. Our topic guide included overarching questions to drive the story telling in our narrative approach. These included military and prior civilian sporting experiences and identity, regimental and service history, disability (medical), disability (social meanings, beliefs and attitudes), broad transition experience, the role of sport and physical activity, Invictus Games experience, training, identity and community, and other aspects of identity and narratives.

We have redacted small regiments, specifics of age, injury and those performing in IG as it would mean they could be easily identified. The breakdown of respondents can be seen in the Table 1 below:

**Table 1:** Breakdown of respondents by age, gender, military transition characteristics and regiments (where sizeable enough role and disabilities will maintain anonymity).

Participant pseudonym	Age; Gender	Sporting background	Military and injury/discharge background
Mike	52; M	Sit-skiing, archery, target shooting, sailing, paddle, and motorcycling PE teacher once leaving the Navy	Navy – served for 16 years as a fighter controller and then joined the Reserves Paralysed in 2003.
Kya	45; F	Pre-injury: fitness instructor, football, cycling, running, triathlons, climbing Post-injury: marathons, triathlons World Record holder for half marathon and full marathon in normal wheelchair	Army – served for one year Chronic Regional Pain Syndrome (CRPS) and concerns about her sexuality
Wendy	55; F	recumbent bike, squash, tennis, swimming, skiing, walking, triathlons	RAF – dental officer since 1990 Voluntarily left has joint hypermobility syndrome and fibromyalgia and PTSD.
Mira	41; F	Main sport was rugby and white-water rafting instructor Now: avoids impact sport – yoga and Pilates	Army – medically discharged from Royal Military Academy Sandhurst heart murmur Neck and back problems that led to a rubber disc replacement.
Andre	30; M	Pre: Boxing teams Always very physically demanding in the Army	Army – infantry soldier Eight years’ service - Collapsed during an eight-mile run admitted to cardiac care unit Medically discharged
Luke	35; M	Prior to injury: triathlons, clay target shooting. PTI course, personal trainer and cross fit coach Post: rowing, cycling, cross-country skiing	Army – served for over 16 years in the Royal Signals 2021 – medically discharged for PTSD and had to undergo reconstruction of arm – now 55% function. Shot multiple times on active duty.
Terry	61; F	Prior: GB triathlon, squash, tennis, hockey Post: Trike, occasionally swims	Navy – Women's Royal Navy Service Served for 6 years. Coerced out of Navy left with a broken back Finds it hard to stay on her feet for extended periods of time.
Lynne	43; F	Prior: swimmer Post: Powerlifting, rowing, swimming, archery, ultra-triathlon, half ironman.	Army – air dispatcher 17.5 years of service. Medically discharged for mental & physical health Army issued boots wore away at Achilles tendon, left with nerve damage and drop foot.

### Theoretical framework

The veterans' stories were critically understood by using the conceptual model and typology from Caddick et al. (6), this can be seen in Figure 1 below. At present there is no theoretical framework specifically available for the transition of veterans through sport and physical activity. This is in part why this theory and associated concepts were used, and in part why it is a good starting point in a novel area. They discovered four dominant narrative types of 'struggling against decline', “minimization”, “victimhood”, and “life-as-normal” throughout the veterans' stories. These types were used as a structure for questions when interviewing the participants in this study. Although some follow the typologies exactly, some narratives are less set to specific types, overlapping and interplaying throughout their stories. An advantage of using a typology is to “render orderly what initially seems merely individual in its variety” (59:46), thus, preserving the complexity of the participants stories. Moreover, using a typology of narratives underlined similarities between the participants stories whilst preserving their different experiences (6). However, the narratives identified should be viewed as the overarching themes of the participants' stories, rather than being perceived as compressed into simplified types. We note Caddick et al's (6; 3) cautionary sense that, “we also consider the narrative types as *relational*, with the fluidity and interplay between them represented by the following conceptual model”. The authors also suggest that we must guard against simplicity and the pigeon holing of stories into such neat themes and categories. For this reason, we use the framing model as an abductive starting point, aware that in reality our starting point is in itself fluid, relational constructed and political.

The theory of ableism (61) refers to discrimination against the individuals with disabilities, often resulting in the privileging of able-bodied individuals. Thus, para-sport is less likely to get the recognition, attention, and support that it deserves (60, 61). This intersects with the experiences of disabled veterans and the typology employed by Caddick et al. (6). For instance, the narrative type of 'struggling against decline' as veterans are coping with physical conditions resulting from their service, highlights the challenges that disabled veterans face in navigating a society that is designed for able-bodied individuals alongside the lack of access to resources and support.

### Analysis

Interviews were recorded and transcribed using *Microsoft Teams* (Microsoft Press Richmond, WA, USA) and compressed into electronic text files. A Voice Tracer was used as a second form of recording and transcription device. The transcriptions were read, compared and corrected against the audio file. These recordings were deleted, and pseudonyms were used to preserve the confidentiality of the participants (57). A narrative analysis method was used to focus on each participants narrative, in story form (56, 57, 62). We employed an abductive research design here moving between data and theory, literature and evidence back and forth. This was preferred to maintain the complex interplay and textured fabric of the participants lives, feelings and their experiences (56) and suits narrative analysis. Similarly, Caddick et al. (6) used narrative analysis to understand what a particular story does, in relation to the teller and the listener, calling interest to the inherently social process by which stories encourage people to be who they are and helps us understand what they do (6]; Reissman). The participants stories were interpreted using a dialogical narrative approach (59), on the premises that the participant is considered as an authority of their own experiences, implying that the interviewer's role is to gain knowledge and learn from them. Thus, recognising the hermeneutic openness and the unfinalisability of the stories told by the veterans (59, 63). However, consideration was taken when using any type of narrative analysis. The stories were generalised and are far more complex than they come across when compressed into several types. Moreover, there is fluidity and interplay between them represented by the following conceptual model (6).

## Research findings

Similar to all narratives, the accounts of disabled veterans were recounted within a broader framework that influenced how they were conveyed. In the analysis of veterans' stories, a typology developed by Caddick et al. (6) was used to identify five narrative types: “struggling against decline”, “minimization”, “victimhood”, “life-as-normal” and “dramatic overcoming”. These narrative types reflect the key themes that underlie the veterans' stories, although some stories may deviate from these types or contain multiple overlapping narratives.

### Struggling against decline

Despite issues including limited mobility or unbearable pain, many veterans feel the need to continue to maintain their independence, whilst 'struggling against decline'. Although Caddick et al. (6) compares the stories of ageing and limb-loss, the struggling against decline narrative is applicable to those in this study as they battle against the decline of their disabilities and impairments, to maintain independence. Engaging in independent challenges and activities can positively impact recovery and well-being, as found by Burke and Utley (4). This struggle for independence occurs in sports and activities where people with disabilities, like those who use wheelchairs, may receive unnecessary assistance due to society's perception of their capabilities. In both cases, individuals strive to maintain their independence despite the challenges they face:

When I was doing the London Marathon, the world record, I had a banner on my back saying “World Record Attempt” and I wasn't allowed any help at all, quite rightly, it's a solo record. I was going up my first hill and I felt a hand on my back, a lady was trying to push me up the hill, I had no choice but to shout you can't touch me.. That could be my record gone, because you're helping me, and I don't need your help. (Kya)

Rather than emphasizing the challenges they faced, numerous narratives focussed on the capabilities of the veterans and the tasks they are still able to perform. Thus, relating to the militarised mindset that veterans have due to their time in the Armed Forces (8). Despite some having to modify their daily tasks to accommodate their disabilities, the veterans expressed a preference for living as 'struggling *against* decline' as independently as they can, without relying on the aid of others:

I have learned to find ways around problems rather than just seeing a problem and giving up. I think the military has helped with the practical leadership tasks that we had to do through training and the concentrating that you see throughout your career. It meant that I just saw each challenge as the next obstacle to overcome and not one that was going to defeat me… you find ways around it. I live alone in a little house, I drive, and I do all my shopping. That's my choice… I'm engaged, have a fiancé with kids… I take the boy; we go off to football. (Mike)

Again, this further emphasis here is aligned to the Caddick et al. (6) conceptualisation and it features of struggling against decline. Grimmell (15) in citing the seminal work on military transition by Brunger et al. (64, 95) proposes,

“the experiences among former service members are categorized within three broad themes: characteristics of a military life, loss as experienced on return to civilian life, and the attempt to bridge the gap between these two lives. Additionally, transition from military to civilian life can be viewed as a shift in sense of self from soldier to civilian”.

As the analysis across our established Caddick model (6) will show nuances by military identities from a sociological perspective offer fresh insights to this established typology. But whilst the three categories are of relevance, military transition and social identity is a highly individualised process (65).

### Minimization

According to Caddick et al. (6), the minimization narrative involves recognising that some veterans seen in this study do not want their injuries and disabilities to be the primary focus of their lives and stories. For example, rather, they chose to emphasise their prosperous careers and achievements after their injuries, emphasising the importance of returning to a sense of normalcy despite their impairments:

Does it affect my life? Yes. Do I let it affect my life? No. (Lynne)

Terry shared that they continued to participate in sport and PA even after injury or leaving the military. Hawkins et al. (44) found that engaging in sports allowed them to alter their self-perception following their injury and look past their disabilities. For many, this was a way of feeling accepted and perceived as “normal”, even if it meant utilising adapted forms of activity. It was heartening for them when people could not recognise their disability, validating their efforts to normalise their lives:

I opted for a trike… it allows me to be normal, whatever normal is… I want to be able to try to hide it (the disability) because I want to do what everyone else can… I'm quite strong and I keep fit… I like people to say, “Can you do that?” and I will have a go. (Terry)

Another characteristic that distinguished minimisation was the focus on promoting equal contribution to society having left the military with a disability and therefore, demonstrating one's competence at the same level as able-bodied individuals. According to Caddick and colleagues' (2014) systematic review, most of the studies indicated that veterans, despite being medically discharged, could lead “fairly normal” lives within the constraints of their physical limitation:

I haven't struggled. I've done pretty well on job interviews and things like that. I've been in employment since I've left the military. I found it difficult, but I've not found it difficult to get a job. (Luke)

These neat boundaries are also challenging using this theoretical model and framework, as we set out at the start of this paper the move away from “broad transition” ideas to individual journeys and narratives. But the conceptual richness of the Caddick et al. (6) framing does align to the stories in this IG focused paper. Furthermore, this narrative is, in itself, a strong contrast to “aborted transition” between the two dichotomous cultures (military and civilian) suggested by some studies (15).

### Victimhood

The victimhood narrative has become an important channel for understanding injuries sustained by military personnel during combat, often leading and assisting their medical discharge. Although controversial, society is focussing on celebrating victimhood, attempting to understand soldiers' experiences and portray them as “normal” (44) through the conceptual development of sympathy towards veterans and amplifying frustration at the Government for permitting victimisation to occur (6). This is closely linked to the notion of being able to the construction of a fulfilling civilian identity after service (55).

During interviews, a substantial number of veterans expressed a deep and real sense of frustration and anger about how they were treated as well as their concerns for the future treatment of veterans after leaving the military. Such emotions felt hard to analyses in a neat conceptual box. But by adopting the stance of being a victim, we can see how individuals can rationalise their challenge to authority, and both advise and request preferential treatment (66). This was often emphasised when listening to how and what the veterans want to see theoretically changed enacted for their lived identities and narratives (6):

I've said if anyone goes through this in the next months or years, be conscious of X… you’ll get to the point easier and not frustrate the individual along the process… Another thing I found frustrating, although I had no choice in being medically discharged, after 16 years, I was a career person, I aimed to stay in the military, but that was taken away from me… I wasn't allowed to join the Reserves. They were telling me that everything that I've worked for over the years, I can no longer have any involvement with. (Luke)

In contrast, however, using sport and PA as a means of recovery and support when leaving the Armed Forces may not always be feasible. In some ways this challenges the very notion of sport frameworks such as Caddick et al. (5) and others before him may conceptualise. A veteran, who was medically discharged, shared his experience of taking legal action in the hopes of seeking justice after being pressured to perform, despite not feeling capable at the time. The individual continues to face challenges with everyday tasks and is no longer able to perform the same level of sport or PA as before:

I told the instructors on the day, as we were running, that I wasn't feeling good, and I was told to “crack on”… I started suing the army. It was more to get the Army to admit what they had done wrong rather than the money side of things. It took a long time to get them to do it… I knew, roughly, the realms of what they were going to pay me out. And it is life changing… The next day I woke up and I was thinking about it… I got emotional because it doesn't matter how much money someone throws at you, that injury is still there. It's never going away. I felt cheated. (Andre)

Andre's narrative portrays a critical sense that sport and PA may not be the vehicle or panacea we perceive to address complex societal and individual transition dilemmas.

### Life-as-normal

The life-as-normal narrative shows some relevance to where the veterans' do not want to share their illness, injury, or traumatic experiences. They wish to preserve the normality that the social world takes away from them, and, subconsciously resent them being treated differently. The life-as-normal narrative seeks to minimize illness. As such, it highlights how societal norms around masculinity (8), such as being physically able, stable and successful, can shape the expectations of disabled veterans:

Big alpha males don't like talking about emotional things, but I found that started to help… mental health nurses then realised the extent of my mental health problems… I'm not usually an emotional person. I took things on, dealt with them and got on with my life… I didn't know how to ask for help as I previously was a person who was at the top of my game… Rehab helped getting back outdoors, switching off the world around me… The biggest driver for me was I want to compete in sport. (Luke)

However, there should be careful consideration when theoretically using this narrative. There should be an understanding as to whether this is how all veterans wish to speak about their past or whether it is just coincidence of the ones selected. Healthy and able-bodied individuals must be careful that they are not choosing to treat the veterans’ lives as normal, to avoid awkwardness (59). Moreover, Frank (59) expressed that the “life-as-normal” narrative presents another issue when people are required to speak about their illness or disabilities. When normality is unsustainable and acknowledgment is required, particularly if the illness could become worse, it is more difficult to communicate if they have been treated as “normal” previously. Many veterans interviewed referred to able-bodied people as “normal” with a large emphasis on the fact that they (the wounded) are not “normal” *per se*:

Guys will say “there's nothing wrong with her” … But then it's easier when it (the disability) is seen… I have had to go through so much monotony and tests and “is it real?”, showing x-rays of my back, or proof I've got to put forward… My word should be enough, but it's not. (Terry)

The very human meanings attached to “normal” living, return to normal, and the process of transition are shown here to be inherently complex. Furthermore, analysis of these narrative stories indicates the challenge of speaking about their disabilities.

### Dramatic overcoming

The IG competitor represents the prevailing contemporary narrative of dramatic overcoming, which has come to epitomise the rehabilitation process which analytically perhaps has most potential for buy in through such research spaces. It has appeal, and respondents talk of such identity components through their stories. These narratives tend to emphasise the athletic feats of injured veterans (5). The cultural prominence of the Invictus narratives has contributed to the widespread adoption of the dramatic overcoming of disability as the preferred mode of storytelling. Previous studies that did not find evidence of dramatic overcoming imply that the facilities and opportunities available to veterans were not as plentiful as they are today. Caddick et al.'s (6) study, which focused on an older age group, revealed that sporting opportunities were less pertinent, accessible, and attractive to veterans, hence the absence of data in the study. In contrast, dramatic overcoming was prevalent in this study, likely because the participants were young and recruited through the IG Foundation page. For them, much like previous research (2, 4, 44, 48), challenge and competitive sports played a pivotal role in their mental and physical recovery:

Eleven months from being injured, I was in a sit-ski…The doctor said I would be in hospital for two years… But it's amazing what the human body can do if the mind suddenly wants to do something… Other people had not found that mental anchor… I said that I would learn to ski and get in the British team and go to the Paralympics, twelve days after being paralysed. So that gave me this drive. It gave me hope. (Mike)

As participants trained towards significant challenges, they utilised sport and PA as a form of self-medication. Research conducted by Brittain et al. (2) found that sport competitors benefited from the competitive and goal-directed rehabilitation, which aided in improving the physical, social, and psychological impacts of their injuries in an engaging and demanding way:

They wouldn't believe that a female could do a marathon using a non-sport wheelchair. I did four marathons last year. I've already done three this year. I'm proving that it is possible and gaining some momentum… It was a case of, I can't just sit here feeling sorry for myself… while I can't wear one (prosthetic leg), if this is going to be the way I can do sport again, then I'll start with what I have. (Kya)

## Discussion

Participation in sport and PA, and events such as the IG, provides veterans with a renewed sense of individual involvement in facing challenges and overcoming their disabilities. Less was spoken of around development of camaraderie and social connection and allowing them to connect with others who have experienced similar challenges. But we suspect much of this may come from the one off event aspect as opposed to the build-up training and “new identity work” outside of IG specifically but closely linked to their military identity (11, 16, 65).

This is particularly relevant when considering Bourdieu's concept (67) of “military habitus”, suggesting that being surrounded by individuals who have undergone similar transitions out of the Armed Forces allows veterans to feel a sense of belonging and gain back from their careers (2, 5–7). By having goal-directed PA, most of the participants have turned to sports as a means of self-medication and to gain independence. This supports findings that sport and PA can function as medicine if there is structure, support, accountability, and inclusivity (4). Despite the recurrent lack of support from the military upon their departure, competing in sports with other disabled former military personnel enabled veterans to maintain their cherished identity. This also aligns with what others have referred to as “aborted transition” where it has been suggested a cultural dichotomy may hinder transition from military to civilian life (12, 13, 15). Clearly, in this paper we are specifically examining the role of sport to address this.

In certain instances, participants who sustained injuries while serving in the Armed Forces and were subsequently medically discharged have reported a significant decrease in their activity levels. As a result, they have felt a sense of emptiness, since sport and PA used to play a leading role in their lives while they were in the military. Hawkins et al. (44) discovered that disabled veterans had lower rating for physical independence and occupation. This implies that while sport and PA might offer some assistance, it is improbable for them to be as effective for disabled individuals as they are for those who are able-bodied. According to McDermott's (68) research, veterans who have acknowledged the conclusion of their military careers and have planned for their transition in advance are more likely to manage the transition successfully. This indicates that they had not been medically discharged and were ready to depart on their own volition, unlike the veterans in this study.

The results gave rise to a narrative typology that Caddick et al. (6) did not follow. This is associated with the “getting on with it” approach and the corresponding mentality that all the veterans from this study proved. One participant repeatedly employed the phrase “cracking on”, comparable to Ledwidge's (69) interpretation of the term as an unyielding, proactive and combative attitude. Similarly, Walker (70) identified a typology of military leavers, ranging from “transformed” to those who were “blighted”. Those described as “blighted” had been negatively impacted by their service, while those who considered themselves to have been positively “transformed” were more common. The “*getting on with it*” attitude that veterans display in their personal and civilian lives can be attributed to this factor. By adopting this military mindset, participants were better prepared for the mental and physical demands that may arise during times of serving, thus reflecting the reality of such conflicts. Moreover, by adopting this approach towards life beyond their military service, into civilian life, veterans have the power to shape their own perspectives on the challenges they will encounter.

The participants in this study refuse to accept the medical model of disability, which views disability as an individual medical problem that needs to be fixed and instead embrace the social model (38, 71). This also advances and develops previous work around veteran identity and military identity which shows that for some of our veterans the “getting on with it” narrative may sit outside some existing psychological models of military identity. Previous research on ableism supports this perspective, as it highlights the obstacles and barriers created by society, environment, and other external factors that disabled individuals face (54). As evidenced by the challenges faced by participants in this study, even small daily tasks like moving on “cobbled pavements” can pose significant barriers for people with disabilities due to the societal obstacles that are often overlooked by able-bodied individuals.

While the theoretical framework proposed by Caddick et al. (6) provided a suitable foundation for the study's findings, it primarily focused on the challenges faced by an ageing population coping with experiencing limb-loss and ageing. In this study, the participants have not yet had to go through the resistance of decline due to ageing (in most cases) and are more faced with challenges of experiencing disability and achieving independence. Lastly, the study was subject to recruitment limits since the participants were solely recruited through the WIS Invictus Games Noticeboard, which could have led to a sample that already had a predisposition towards sport and PA, given their interest in IG.

## Conclusions

The findings of this study revealed novel, evocative, and insightful portrayals of veterans' first-person perspectives of disability and how sport and PA has impacted their transition into civilian life, through a dialogical narrative approach. This extended previous research by developing a nuanced understanding of how veterans' experiences since injury affect their wellbeing, independence, and future, after leaving the military. Engagement in sport and PA permitted individuals with disabilities to reconcile themselves with certain aspects of their new identities, in accordance with previous findings (2, 44, 70). According to the participants, gaining independence and learning a new normal was a crucial part in accepting their future. For an event spanning 23 countries and with global scale events research into this area is minimal (11, 65, 72). The exception to this is a quantitative study by Forces in Mind (72) spanning the impact of IG Sydney 2018 and The Hague Games of May 2021. We hope that in this qualitative study we complement existing insight into the program design to aid, rehabilitate and support military veterans.

Secondly, the reconnection with other military personnel in a comparable situation to them allowed them to be around other people who they felt would understand them and the way they were feeling, as well as reconnecting them with their cherished military identity. Such military identity must be understood from a complex nuanced and contested empirical and theoretical position (6, 15, 16, 65). Sporting events like the IG provided veterans with a renewed sense of camaraderie and social connection, allowing them to connect with others who have experienced similar challenges. Thirdly, there was an overcoming a sense of victimhood, proving themselves and observers that they still held “worth” and could achieve things by overcoming challenges that even normal people found difficult. This is particularly relevant to the intersectional themes around ageing and the IG. As stated above in the discussion, one of the participants views themselves very much as an active part of their sport, but for others in society and IG events in the future this may be perceived differently as they are 61 years old. Such socially constructed perceptions of military transitions linked to age, gender, disability and other intersectional aspects (such as regiments, armed forces divisions and sexualities for example) are key.

We also acknowledge that the study being qualitative in nature focuses on the rich, textured personal life histories of individuals who (partly) transitioned through IG. Being UK ex-services in focus is a limitation and future insight from the other 22 partner nations would be significant. IG is also well funded, globally promoted and a one-off large scale event. Local military veterans in the UK account for 2.7 million people (65). Not all are disabled, but do require support, such studies require wider consideration of the role of sport.

This study has several novel concluding implications. Promoting sport and PA and providing opportunities for veterans to participate in sporting events can be a valuable tool in supporting the transition of military veterans with disabilities into civilian life. It can provide a sense of belonging, improve mental and physical health, and essentially, facilitate the process of redefining identity after leaving the military. Moreover, this study helps to highlight that an alternative approach should be taken to help those who are not able to partake in sport and PA, to ensure additional support and options, and to prioritise the care for all veterans (11, 65). It may help in the planning of expanding field of UK and other military transition programmes (73, 74).

Future research should focus on developing effective interventions that promote sport and PA and support veterans in their transition into civilian life. Sensitivity is important when approaching interviews in terms of methodology, as it allows for validation of the responses provided by veterans. This approach helps to preserve the integrity of the data while delving into the complexity of the veterans' lives (56, 75). With a growing body of work in the field of military veteran education, sport, health and exercise linked to rehabilitation (5, 6, 11, 15, 65, 72, 76) it appears that a consolidated new agenda for qualitative research is now emerging alongside the more medicalised model-led that sits within the quantitative field of methodological health, psychology and medical enquiry. We hope that what our work has done is open the diversity of voices in this space beyond narrow pre-conceptions of who, what and where the veteran represents and the multitude of paths that sport and PA can open for such communities to engage in transition into civilian life. But equally we hope to have encouraged a more interdisciplinary examination of this little understood global social and sporting event phenomenon and opened a new research agenda in the Invictus Games space around military transition, military identities and new ways of looking at narrative typologies that do exist (6, 16, 65).

## Data Availability

The datasets presented in this article are not readily available because data has links to individuals military historical records. This restricts their availability. They are anonymised as a result and references to their service records removed apart from broad non-identifiable sections (Navy, Army and so on). Requests to access the datasets should be directed to c.mackintosh@mmu.ac.uk.
